# Effects of community health volunteers on infectious diseases of children under five in Volta Region, Ghana: study protocol for a cluster randomized controlled trial

**DOI:** 10.1186/s12889-016-3991-z

**Published:** 2017-01-19

**Authors:** Yeonji Ma, Heunghee Kim, Yinseo Cho, Jaeeun Lee, Joseph Kwami Degley, Abdul-Ghaffa Adam, Gyuhong Lee, Hoonsang Lee, Seungman Cha

**Affiliations:** 1 Korea International Cooperation Agency, 825 Daewangpangyo-ro, Sujeong-gu, Seongnam-si, Gyeongi-do Republic of Korea; 2 Korea International Development Institute, 92 Saemunan-ro, Jongno-gu, Seoul Republic of Korea; 3 Ketu South Municipal Health Directorate (Ghana Health Service), P.O. Box 126, Aflao, Volta Region Ghana; 4 Devtplan Consult Limited, 212 Lame Dwaahe Street, Adenta-Accra, Ghana; 5 Department of Disease Control, Faculty of Infectious and Tropical Disease, London School of Hygiene & Tropical Medicine, Keppel Street, WC1E 7HT London, UK

**Keywords:** Community health workers, Community health systems, Diarrhea, Malaria, Child health, Ghana

## Abstract

**Background:**

In many low- and middle-income countries, community health volunteers (CHVs) are employed as a key element of the public health system in rural areas with poor accessibility. However, few studies have assessed the effectiveness of CHVs in improving child health in sub-Saharan Africa through randomized controlled trials. The present study aims to measure the impact of health promotion and case management implemented by CHVs on the health of under-5 children in Ghana.

**Methods/Design:**

This study presents the protocol of a cluster-randomized controlled trial assessing the impacts of CHVs, in which the community was used as the randomization unit. A phase-in design will be adopted, and the intervention arm will be implemented in the intervention arm during the first phase and in the control arm during the second phase. The key intervention is the deployment of CHVs, who provide health education, provide oral rehydration solutions and zinc tablets to children with diarrhea, and diagnose malaria using a thermometer and a rapid diagnostic test kit during home visits. The primary endpoints of the study are the prevalence of diarrhea and fever/malaria in children under 5 years of age, as well as the proportion of affected children receiving case management for diarrhea and malaria. The first and second rounds of household surveys to collect data will be conducted in the first phase, and the final round will be conducted during the second phase.

**Discussion:**

With growing attention paid to the roles of CHVs as an essential part of the community health system in low-income countries, this study will contribute valuable information to the body of knowledge on the effects of CHVs.

**Trial registration:**

ISRCTN49236178. (June 16th, 2015)

**Electronic supplementary material:**

The online version of this article (doi:10.1186/s12889-016-3991-z) contains supplementary material, which is available to authorized users.

## Background

Despite the substantial progress made between 1995 and 2015, over half of the deaths in children under 5 years of age in sub-Saharan Africa are still caused by malaria, diarrhea and pneumonia [1]. Malaria alone caused 306,000 deaths among under-5 children in 2015 globally, including 292,000 deaths in sub-Saharan Africa [2]. Diarrhea, largely due to the lack of safe water, sanitation and hygiene, killed 578,000 children in 2013, accounting for 9.2% of under-5 child deaths worldwide [3].

Of the 62,000 deaths among Ghanaian children under 5 years of age in 2013, the majority were caused by malaria (20%), acute respiratory infections (13%), and diarrhea (8%) [4]. While other infectious diseases, such as HIV/AIDS and measles, have significantly decreased over the last decade in Ghana, diarrhea-specific child mortality has stagnated and malaria-specific deaths have decreased only by one percentage during the same period [4].

Globally, given the limited human resources in the health sector, a community-based approach has been promoted as a cost-effective and pro-poor intervention to improve the accessibility of health care [5, 6]. Community health workers (CHWs) are often employed as a key element of the community-based approach to the rural population of low- and middle-income countries [7]. CHWs, in general, are defined as the non-professional lay health workers who are originally from the community and equipped with training and incentives to provide promotional, preventive, or curative health care services to the community members [8–10]. They are known by various names in different settings, depending on the type of services offered, the compensation scheme, and the level of integration with the formal health system. For instance, Nigeria administers a village health worker program in which they are allowed to perform promotional work only, whereas health extension workers in Ethiopia are allowed to offer curative services for major illnesses. Lady health workers in Pakistan are paid as government employees, whereas a community health worker program in Tanzania operates based on volunteerism without regular payments [11].

In Ghana, the Community-based Health Planning and Services (CHPS) initiative was adopted in 1999 as a national policy establishing a community health system to improve the accessibility and affordability of health care for residents in the remote areas [6]. A distinctive feature of the CHPS system is the requirement of strong community engagement and participation. Community health nurses (CHNs) and community health volunteers (CHVs) are considered to be CHWs in the Ghanaian CHPS system. CHNs are formally trained for 2 years, bear the main responsibility for community health management within the formal governmental apparatus, and are on the government payroll. In contrast, CHVs are trained for a week and voluntarily support the outreach activities of CHNs and community participation, and are not paid by the government. Since community participation is a key aspect of the mechanism of the CHPS system, the role of CHV is as much critical as that of CHNs [6, 12].

A number of studies have demonstrated that community-based health systems, particularly those involving CHWs, have led to improved access to maternal healthcare services [13–15], modern contraception [16, 17], and neonatal care among the poor [18–22]. However, recently only few studies have investigated the impact of interventions made by CHWs on childhood illness in sub-Saharan Africa, using randomized controlled trials (RCTs) [23–26]. Moreover, many previous studies [27–31] have evaluated the effect of CHWs only with the curative treatment while only one [26] testing the effect with the preventive roles with RCT. A recent Cochrane systematic review evaluated 10 trials investigating the impact of CHWs with the expanded roles including prescription of anti-malarial drugs on the health of African children [32]. Eight of them demonstrated the prescription of the drugs by CHWs without confirmation despite the World Health Organization’s recommendation to perform malaria diagnostic testing on all suspected malaria cases before administering treatment since 2010 [2]. To our knowledge, only one study published using RCT so far has tested the impact of CHWs in diarrhea management without provision of antibiotics while many others include the intervention with antibiotic prescription [26].

Therefore, there is need for research testing the impact of CHWs, specifically regarding the promotive and preventive roles in major childhood illnesses in sub-Saharan Africa.

The study aims to measure the impact of CHVs on reducing childhood illnesses, especially malaria and diarrhea, in Ghana, which is one of the countries that has implemented a CHW program. We plan to explore the impact of CHVs, especially with regard to their role in the provision of health promotion, first-aid management for diarrhea with oral rehydration solution (ORS) and zinc tablet, early diagnosis of malaria using rapid diagnostic tests (RDTs), and referrals for further treatment.

## Methods

### Study setting

**Fig. 1:**
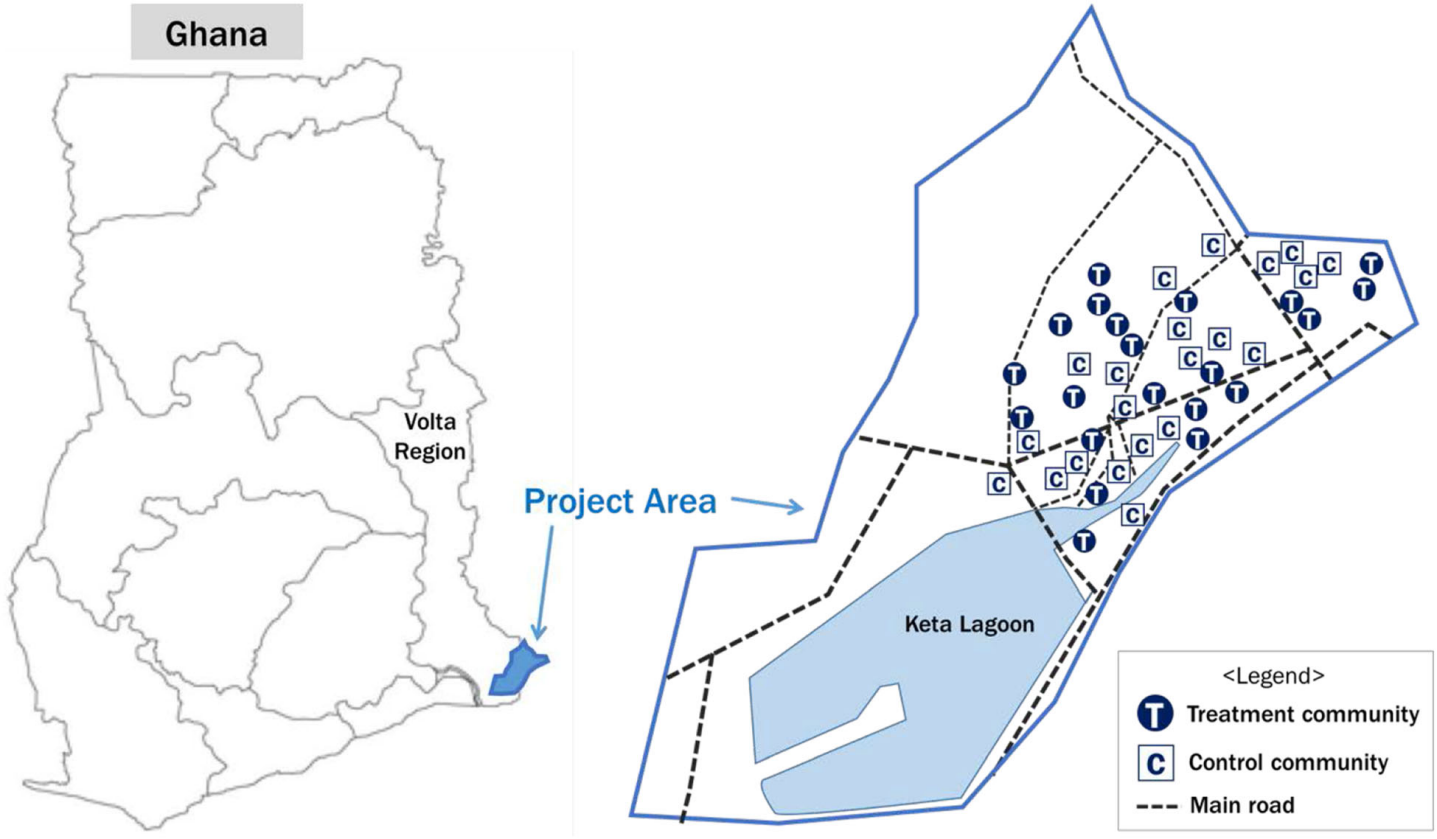
Study area

The Ketu South district is one of the 25 administrative districts in the Volta Region, located in the southeastern corner of Ghana (See Fig. 1). The Ketu South district is a relatively low land area, with altitudes ranging from less than 15 m at the coast to 66 m inland. The 2010 Population and Housing Census recorded a total population of 160,756, with 52.9% of the population consisting of females. The projected population for the district for 2015 based on an annual growth rate of 2.5% is 181,881, with 36,376 children under 5 years of age. The people of Ketu South are the Ewe tribe, which inhabits some parts of Togo, Benin, and the Volta Region of Ghana. They are a patrilineal society governed by a hierarchical, centralized authority. The district is culturally homogenous, with negligible variations. According to the 2010 Population and Housing Census, Christianity accounts for 59.0% of the total population, followed by traditional religion (27.9%) and Islam (3.5%). The people living in the district are predominantly fishermen, fish mongers, petty traders, and weavers of *kente* (a specific type of silk and cotton fabric that originated in the Ashanti Empire), with a few government workers interspersed among the population.

### Study design

In the study, the community was taken as the randomization unit, because we expected that CHV activation will impact disease prevention and treatment among households within a given community, which is where people interact with one another most closely. All interventions related to CHV activation will be performed at the level of each community. A phase-in design will be used for the study. CHVs will be activated in the intervention arm during the first phase, and a comparable intervention will be performed in the control arm during the second phase.

The study was approved by the Ghana Health Service Ethics Review Committee under the Ministry of Health, Republic of Ghana (Ethics approval ID: GHS-ERC:07/01/15), and registered as an international standardized RCT on June 16, 2015 (ISRCTN49236178/http://www.isrctn.com/ISRCTN49236178).

### Primary endpoint

This study aims to assess the effect of CHVs on multiple child health outcomes. The primary endpoints of the study are the prevalence of diarrhea and fever/malaria among children under 5 years of age, and the proportion of children receiving case management for diarrhea and malaria. We plan to use the 7-day prevalence of reported diarrhea and the 14-day prevalence of fever/malaria in the household as indicated in caregiver’s reports.

For the case management for diarrhea, we will investigate whether a child with diarrhea has been administered ORS. For malaria, we will measure whether a child with malaria symptoms was tested using an RDT kit and whether he or she was referred to a health facility if diagnosed with malaria.

The survey will be conducted three times at the household level: the first and second round will be carried out in the first phase, and the final round will be carried out in the second phase.

Diarrhea is defined as 3 or more instances of watery stools within 24 h over the last 7 days. Febrile episodes will be assessed as a proxy indicator of malarial prevalence based on caregiver's reports.

The main outcomes and indicators are presented in Table 1.

**Table 1:** Main outcomes and indicators

Indicator (%) (Denominators are within the respondents of the household survey)		
Use of ORS among under-5 children with diarrhea	Numerator	Number of U5C^a^ who took ORS^b^ when experiencing diarrhea over the past 7 days
	Denominator	Number of U5C who experienced diarrhea in the past 7 days
Prevalence of diarrhea in under-5 children	Numerator	Number of U5C who experienced diarrhea in the past 7 days
	Denominator	Number of U5C
Diagnosis rate of malaria in under-5 children using an RDT	Numerator	U5C diagnosed with RDT for malaria in the last 2 weeks
	Denominator	U5C who have complained of fever in the last 2 weeks
Malaria incidence in under-5 children	Numerator	U5C who have complained of fever in the last 2 weeks
	Denominator	Number of U5C

^a^ *U*5*C* under-5 children, ^b^ *ORS* oral rehydration solution

### Intermediate indicators

Intermediate indicators for the prevalence of diarrhea in children include the hand-washing behaviors of caregivers at 4 critical times (before cooking, after defecating, before feeding the child, before eating). An intermediate indicator for child malarial prevalence is the utilization of insecticide bed nets the previous night while sleeping.

### Process indicators

We are going to carry out a process evaluation along with the impact evaluation to investigate dose delivered and dose received. First, logbooks will be recorded by CHVs themselves and signed by caregivers, where we can assess how many key messages have been delivered at each household level. Second, four rounds of household survey will be conducted, with which we will measure how many and what key messages caregivers could recall.

### Sample size calculation

Based on a preliminary survey in 2013, we estimated that the malaria prevalence was 25% and assumed that the prevalence would be reduced by our intervention by 25%, based on systematic reviews [33]. Assuming a coefficient of variation of 0.5, a 20% loss to follow-up, a study power of 80%, a 25% prevalence of malaria in the absence of an intervention, and a 25% relative decrease after the intervention resulted in the need for 40 clusters and 950 children per arm, using a formula published elsewhere [34].

### Household sampling methods

**Fig. 2:**
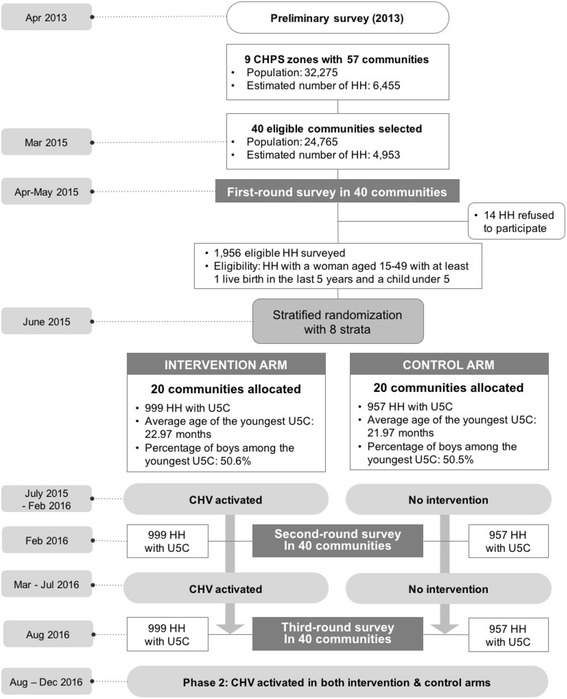
Flow diagram of the trial

The demographic information of each community in the Ketu South district was compiled by the health directorate through reports by CHNs in each CHPS zone prior to the baseline survey. The total number of communities under the nine selected CHPS zones in the Ketu South district was 57, and we applied the probability-proportionate-to-size method to select 40 communities (See Fig. 2). We excluded urban area for the trial since CHV may operate differently between urban and rural areas.

### Eligibility criteria and randomization

Forty communities were assigned into 8 strata on the basis of the prevalence of diarrhea and economic status, based on the results of the first-round survey. To evaluate the socioeconomic status of a community, we used the proportion of households with wattle and daub houses as representatives of the lowest economic level. We ensured that an equal number of communities within all 8 strata were randomly allocated into the intervention and control arms, with each arm including 20 communities. Microsoft Excel version 15.22 was used for randomization. We ensured comparability in the main outcomes between the intervention and control arms by strictly applying randomization. After conducting the first-round survey, we classified the clusters into strata by primary endpoint and socioeconomic status. In doing so, we were able to ensure a high comparability, and therefore were able to avoid selection bias. Control communities are allocated by randomization among the target communities. We found control and intervention communities well balanced in terms of caregivers’ demographic and socioeconomic status, and child demographic status and several key variables related to primary outcomes of the intervention (Table 2).

**Table 2:** Results of the first-round survey in the intervention and control arms

Variables		Intervention		Control	
		N	% or mean (SD^b^)	N	% or mean (SD)
Caregiver’s age (years)		999	29.00 (7.16)	957	28.86 (6.78)
Religion of respondent	Christian	614	61.50%	599	62.60%
	Traditionalist	280	28.00%	256	26.80%
Education level of respondent	Graduated basic school	188	23.6%	165	22.5%
	Dropped out of basic school	455	57.2%	406	55.3%
	Graduated secondary school	19	2.4%	24	3.3%
Possession of NHIS^a^ Card	Have a valid card	332	33.2%	328	34.3%
Age of the youngest child under-5 child (In months)		989	22.97 (15.95)	946	21.97 (15.94)
Youngest child’s sex	Male	505	50.6%	483	50.5%
Main source of drinking water for the last-born child	Hand-dug well	268	26.8%	248	25.9%
	Sachet water	202	20.2%	223	23.3%
	Borehole	190	19.0%	141	14.7%
	Bottled water	163	16.3%	150	15.7%
	Public standpipe	70	7.0%	96	10.0%
Possession of Child Health Record Book		689	69.0%	671	70.1%
Most recently measured weight of the last-born child	Average weight (kg)	689	9.14 (2.60)	671	8.92 (2.66)
Diarrhea incidence in the last-born child		175	17.5%	192	20.1%
Provision of ORS to the last-born child having diarrhea		94	53.7%	97	50.5%
Febrile episode in the last-born child		294	29.4%	337	35.2%
Child had malaria test when febrile		55	17.7%	57	16.9%

^a^ *NHIS* national health insurance scheme, ^b^ *SD* standard deviation

### Eligibility

Households with one or more under-5 children were eligible for the trial. Households were excluded from the first-round survey if a caregiver declined to participate in the survey. Enrolled households were registered with a distinctive identification code at the time of the first-round survey and will be followed for the next 12 months of the trial period.

### Key intervention

Our key intervention is to activate CHV activities, and the intervention period is 1 year. The main duty of CHVs is to pay regular visits to 40 assigned households in their community and to carry out key activities mainly targeting children under five and caregivers. CHVs are required to visit 20 households every month so each participating family can receive a visit every other month. During home visits, CHVs conduct health education with key messages (Additional file 1) on sanitation and hygiene to prevent diarrhea and other infectious diseases such as hand-washing, management of diarrhea using ORS or recommended home-maid fluid, prevention of malaria including utilization of insecticide-treated nets, nutrition, breastfeeding and so on. CHVs also provide services for under-5 children to manage diarrhea with ORS and zinc and to diagnosis malaria using a thermometer and an RDT kit. CHVs will be requested to deliver all key messages during their home visits.

CHVs will be nominated by community committees on the basis of their literacy, volunteerism, and working experience as a volunteer in health-related fields with government, donor agencies, or non-governmental organizations. The selection criteria are based on the national CHPS guidelines, and the District Health Management Team (DHMT) coordinated the selection process.

The training curriculum for CHVs will be developed in reference to CHV’s training manual [35], as recommended in the CHPS implementation guidelines. District and regional health staff will provide 5 days of training. During the 5-day training program, CHVs will learn about the concept of CHPS, their roles and responsibilities, communication skills with community members, and will gain a basic knowledge of essential maternal and child health care services as well as reporting and recording skills. After the initial training, two types of refresher training will be given. One will involve monthly meetings at the CHPS compounds with flexible topics based on the guidlines and the CHV’s request, and the other is a workshop to be conducted every 6 months.

A backpack, t-shirts, an identification card, a CHV certification, a digital thermometer, and stationery will be provided to CHVs. Health equipment such as ORS packets, zinc tablets, and RDT kits will be dispensed in a timely manner to assist their activities. Limited material incentives of 8.7 USD (30 CHC) worth of food items and 1.5 USD (5 GHC) worth of airtime will be also provided every month. *(The exchange rate on August 1, 2015 was applied: 1USD = 3.46 GHC.)*

The first phase, in which CHVs will be deployed in the 20 treatment communities, will last for 1 year.

We referred to the suggested roles in the latest CHPS implementation guidelines (draft version) and the recommended training manual for CHV training [35] in order to specify the roles of CHVs. Table 3 summarizes the responsibilities of CHVs in the trial. The key role of CHVs is to perform home visits to assigned households in their village, and they are also required to support the community health officers (CHOs) and CHNs, to surveil major health events, to mobilize community members, and to comply with regular supervision and training.

**Table 3:** Job description for community health volunteers

Category	Descriptions
1. Home visit	• Pay a home visit (minimum) once per every two months to 35 assigned households • Provide health education to the mothers and family members through constant delivery of key messages mainly regarding the prevention and management of child illnesses, family planning, hygiene and sanitation, antenatal care, postnatal care, and skilled delivery • Provide diagnostic services in malaria to under-5 children and pregnant women using a thermometer and RDT^a^ kit • Provide ORS and zinc tablets for the primary management of diarrhea in under-5 children • Provide Aquatabs to households without an appropriate source of drinking water • Provide family planning counseling to key decision-makers in the family • Support healthy behaviors of the community members (i.e. help setting ITNs^b^, referring clients to a corresponding health facility)
2. Support for CHOs/CHNs	• Support child welfare clinic (CWC^c^) of CHOs^d^/CHNs^e^ in outreach and mobilize the community • Support CHOs/CHNs in referral and management of clients
3. Surveillance	• Record logbook • Record Community Register for Vital Health Events • Find and report pregnancies and newborn cases
4. Community mobilization and sensitization	• Participate in the community meetings and committees and raise health issues • Organize health talks at community gatherings
5. Reporting and training	• Attend an initial training session for 5 days • Attend 6-month workshop sessions • Participate in monthly meeting at CHPS^f^ and report on monthly activities (a refresher session is planned as a part of the monthly meeting) • Manage the provided supplies and submit a report regarding their use

^a^ *RDT* rapid diagnostic test, ^b^ *ITN* insecticide-treated net, ^c^ *CWC* child welfare clinic, ^d^ *CHO* community health officer, ^e^ *CHN* community health nurse, ^f^ *CHPS* community-based health planning and services

It is critical for this trial to ensure that CHVs are properly activated in the intervention communities. The key indicators for measuring the activation level of CHVs are the frequency of their home visits, their participation in the monthly meetings, and their retention rate (Table 4).

**Table 4:** Indicators for measuring the activation of community health volunteers

Component	Indicator	Measurement tool
Service delivery	The percentage of CHVs who have visited all the assigned households (20) in the last month (among those retained)	CHV logbook at monthly meeting
Participation in monitoring	The percentage of CHVs who participated in the monthly meeting at CHPS more than two-thirds of the whole year	Monthly meeting minutes
Retention	The percentage of CHVs retained	CHO records on CHV supervision

### Project implementation

The Improving Maternal and Child Healthcare project in Volta Region of Ghana is being conducted in the Keta, Ketu South and Ketu North districts from January 2014 to December 2016. The three project districts have a total of 24 CHPS facilities within 132 CHPS zones, with a population of 461,939, covering 92,387 children under the age of 5 years. This project aims to enhance the accessibility, availability, and quality of the health services for mothers and under-5 children. In order to accomplish this goal, it provides equipment to health facilities, deploy and activate CHVs, and promotes public awareness.

The project is funded by the Korea International Cooperation Agency and implemented by a consortium of the Graduate School of Public Health, Yonsei University and the Korea International Development Institute.

### Data analysis

Intention-to-treat analysis will be conducted to explore the effect of CHV activation on childhood infectious disease of under-five children. For diarrheal prevalence as a dependent variable, water quantity and quality, hygiene behavior of caregivers, presence of improved latrines, characteristics of the enrolled child such as age and sex, household income, and caregiver’s education level will be controlled for in quantifying the net effect of CHVs. For malaria prevalence as a dependent variable, the enrolled child’s characteristics, household income, and caregivers’ education level will be controlled. Environmental risk factors such as seasonality and altitude are also considered to be independent variables for malaria prevalence. However, the intervention and control arms in our study site present no difference in those factors so there will be no control for environmental factors. When quantifying the net effect of CHVs on child malaria prevalence, insecticide-treated bed net utilization will not be controlled since it is considered an intermediate factor between intervention and disease outcome. Generalized estimating equations will be used for investigation at the cluster level. The random effects model will be used, taking account of between-cluster variation by assuming that there are cluster-level effects. Per-protocol analysis will be performed to obtain additional background information if the overall percentage of CHVs activated does not reach 80%. Multi-level analysis will also be conducted to explore whether the effects of CHVs on child diarrheal and malaria prevalence vary by community depending on a community’s overall coverage of activated CHVs.

## Discussion

This study seeks to assess the effects of CHVs on the health gains of children under 5 years of age. We thus rigorously applied a randomization process after calculating adequate cluster and household sample sizes.

In this study, we place an emphasis on the preventive role of CHVs in child health. The role of CHVs is to provide education about hand-washing, breastfeeding, the utilization of insecticide-treated nets, ORS treatment and rapid malaria tests, as well as making referrals for sick children to health facilities. We expect the health education using key messages during home visit will facilitate their function for disease prevention. Considering the context of resource-poor settings, the public health implications of the preventive role of CHVs are not trivial.

Previous studies did not describe the nature of CHV training or the availability of further education after the initial course. In this trial, CHVs are trained 1 week at the start of the project and regular training will be provided on a monthly basis by CHOs and CHNs.

We performed a pre- and post-test right before and after the CHV training. If we found a CHV not achieving a score of 80 out of 100, we retrained him or her. To standardize the regular training, standard operating procedure on the implementation of monthly meetings (Additional file 2) will be provided to the supervisors of the CHVs and it includes the guidance on the training. The regular training will be conducted based on the topics and messages from the home visit booklet (Additional file 3) that CHVs were provided with to facilitate their home visit education. Also, the monitoring team, composed of the district health directorate members and the project manager and staff, who oversee the monthly meeting and training activities of all CHVs, will coordinate the contents and quality of regular training through active review and discussion.

The intervention is not restricted to health education. According to the operational definition of this trial, the key intervention is to help CHVs perform their duties, which mainly involve visiting at least 20 households each month to deliver key messages and provide basic health services established by the policy such as malaria diagnosis and ORS provision.

Through in-depth discussion with government officials and community leaders during project design, we realized that merely educating CHVs was not sufficient to help them perform their tasks. We thus developed active supervision and support from government officials such as CHPS in close collaboration with DHMT. By employing a phase-in design for the trial, the government made an agreement that CHV activation would be delayed in the control arm during the first phase since it could be carried out during the second phase. In this regard, we believe that contamination will not take place severely.

No monetary subsidies will be provided to CHVs during the trial to strictly comply with the policy of the Ghanaian government, according to which CHVs function as a bridge between community members and CHNs “without affecting the national wage bill,” and rely on the discretion of community members.

One of the strengths of this trial is that a process evaluation will be conducted in parallel with the impact evaluation. Few studies have investigated the impact of CHWs using process indicators. In this study, we will use process indicators at each stage to assess reliability, services delivered, services received, participation, recruitment, retention and quality. A range of data will be collected and analyzed to evaluate each process: the appropriateness of selection of CHVs, the quality of their training, the frequency of their visits to each household every 2 months, the number of key messages delivered to each household, the number of key messages caregivers can recall, community participation in relation to CHV activities, their retention rate, and changes in preventive and health-seeking behaviors. Implementation of process evaluation will ensure that we explore how the entire health system affects the activation of CHVs in terms of supervision, logistics, incentives, and by what mechanism this occurs (Additional file 4). Four rounds of household survey will be administered to collect data regarding the project process, where we will assess the extent to which CHVs will be supported by the community nurses and the entire health system as a whole.

One of the limitations of this trial is that malaria cases are not being confirmed using laboratory tests. We will measure fever reported by a caregiver as a proxy indicator of malaria prevalence in both arms of the trial. Although RDTs will be used to test for malaria in children with a fever, this will only be the case for households within the intervention arm since the testing will be conducted by CHVs. Despite its seasonal variance, fever, as the primary manifestation of malaria in young children, can be utilized as an indicator of malaria prevalence, although some source has suggested that it must be interpreted with caution [36].

The project team must start recruiting CHVs in control arm during the second phase and also reached an agreement with the local governments to commence CHV activation without any further delay right after the first phase regardless of any pronounced effects on children. If we have to implement CHV activation in the control arm, we cannot maintain two different arms with and without intervention, which means we cannot compare the outcomes of the intervention during this phase. Hence, we ruled out an optimization phase. We thus cannot consider an optimization phase for this trial although we think optimization is important.

Due to the absence of demographic data for the study district, we used estimated population figures for each community derived by the officials of the Ghana Health Service. However, during the baseline survey we found discrepancies between the estimated and actual number of households in communities, and these figures were adjusted during the sampling process in some communities. Although a pre-survey listing the households in each community is recommended to enhance the accuracy of household sampling, budgetary constraints prevented us from carrying out such a survey.

CHWs are expected to offer quality health services to the population at a reduced cost. To that end, CHWs have been often required to perform extensive duties, such as health extension workers in Ethiopia or CHWs in Kenya [11, 37]. However, the quality of care may be harmed when CHWs are overloaded or given complicated tasks with only limited training [37]. CHVs in Ghana are voluntary CHWs who are given a short period of training and a limited range of responsibilities, focusing on promotional and preventive health services. Therefore, this study exploring the effects of CHVs on child health will provide useful information regarding the adoption of voluntary CHW programs in sub-Saharan Africa.

As incased attention has been paid to the role of CHWs in community health systems in low-income settings, the roles, incentives, and training program involved have become increasingly diversified, which hinders a clear assessment of the effects of different types of CHW programs. [10] This study will be a step forward in specifically testing the effectiveness of voluntary CHW cadres.

## Additional files

Additional file 1: CHV key messages. (DOCX 99 kb)
Additional file 2: Standard of procedures. (DOCX 172 kb)
Additional file 3: Booklet for CHVs. (DOCX 8322 kb)
Additional file 4: Logical framework. (DOCX 24 kb)
