# Supplementary material for: Effects of community health volunteers on infectious diseases of children under five in Volta Region, Ghana: study protocol for a cluster randomized controlled trial
Source: BMC Public Health. 2017 Jan 19;17:95. doi: 10.1186/s12889-016-3991-z (PMC5244532; doi:10.1186/s12889-016-3991-z)
Supplement: Additional file 1: — CHV key messages. (DOCX 99 kb) [file 12889_2016_3991_MOESM1_ESM.docx]

**Key messages (CHV home visit)**

(1) Family planning

- Family planning prevents unwanted pregnancy.
- Go to health facility for counseling to find the best choice for each woman.
- Condoms can be always used alternatively.

(2) ANC

- Visit the health facility and attend ANC when you get pregnant.

(3) SBA

- Delivery at health facility keeps you and your baby safe and healthy.

(4) PNC

- Visit the health facility with your baby to attend PNC immediately after an unexpected delivery in the house.

(5) Exclusive breastfeeding for 6 months

- To keep your baby healthy, practice exclusive breastfeeding for 6 months.

(6) Prevention of diarrhea in under-5 children

- Proper hand-washing and clean latrine prevents diarrhea.
- Mothers must wash their hands with soap under running water at 5 critical moments: 1)before cooking, 2)before eating, 3)before feeding, 4) after handshaking, 5)after defecating (visiting the toilet).
- Avoid open defecation and keep the latrine clean to avoid flies.

(7) Management of diarrhea

- Use ORS or Zinc tablet^[[1]](#footnote-1)^ and immediately go to the health facility for treatment.
- If ORS is not available, use rice water, coconut water or mashed kenkey.

(8) Prevention of malaria

- Every person, especially pregnant woman and child under-5 should sleep under ITN(Insecticide-treated net).
- Pregnant women should take SP(IPT) to prevent malaria.
- People diagnosed positive to malaria should go to health facility for treatment.

(9) Prevention of anaemia

- Eat green vegetables and fruits.
- Visit health facility to take iron drugs.
- Deworm your children every 3 months.

(10) Participation in CWC (Child welfare clinic)

- Go to CWC every month for weighing and vaccinating your children up to 5 years.

1. ORS and Zinc? ORS or zinc? [↑](#footnote-ref-1)
