# Supplementary material for: Effects of community health volunteers on infectious diseases of children under five in Volta Region, Ghana: study protocol for a cluster randomized controlled trial
Source: BMC Public Health. 2017 Jan 19;17:95. doi: 10.1186/s12889-016-3991-z (PMC5244532; doi:10.1186/s12889-016-3991-z)
Supplement: Additional file 2: — Standard of procedures. (DOCX 172 kb) [file 12889_2016_3991_MOESM2_ESM.docx]

**
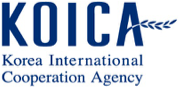

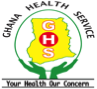
**

**<CHV monitoring guidelines for CHO/N>**

***Monthly meeting and reporting***

1. Hold a CHV monitoring meeting during the first week of every month.

2. The meeting date should be decided considering the attendance of all CHVs.

3. The monitoring record and meeting minutes should be submitted to the municipal health directorate within 3 days after the meeting every month.

4. Monitoring schedule should be notified to the municipal health directorate prior to the meeting.

***Supervision during the monthly meeting***

1. The attendance/lateness of each CHV to the meeting should be recorded and reflected to the incentive provision according to the rules.

2. During the monitoring, CHO should review each CHV/s logbook and record them into the monitoring book. If the log seems insufficient, CHO should direct the CHVs to revise it during the meeting.

3. Home visit activity should be closely monitored: if the CHV visits 20 HH every month, if the CHV isn’t visiting too many HHs per day etc.

4. CHO should prepare for the refresher session every month on at least one topic. The topic can be chosen based on the CHVs’ or communities’ needs or CHO’s judgment. CHO should make sure that its activity should be clearly recorded in the meeting minutes by the secretary CHV.

5. Incentive items and supplies should be distributed in every meeting with the help of CHVs. Incentive items should be given based on the performance each month.

6. CHO should notify CHVs on the CWC schedule of the month and other important schedules and issues.

***Supervision before/after the monthly meeting***

1. CHO should monitor CHVs’ home visit activity. Home visit follow-up on each CHV is recommended every two months.

2. CHO should contract CHVs prior to outreach CWC so they mobilize the mothers.

3. CHO should call or meet with each CHV at least once every month after the monthly meetings for follow-up and close supervision.

4. CHO should give a full support to CHVs when there is a referral case or a rational request.

5. CHO should request for support to municipal health directorate whenever it’s needed.
