# Supplementary material for: Effects of community health volunteers on infectious diseases of children under five in Volta Region, Ghana: study protocol for a cluster randomized controlled trial
Source: BMC Public Health. 2017 Jan 19;17:95. doi: 10.1186/s12889-016-3991-z (PMC5244532; doi:10.1186/s12889-016-3991-z)
Supplement: Additional file 3: — Booklet for CHVs. (DOCX 8322 kb) [file 12889_2016_3991_MOESM3_ESM.docx]

Community Health Volunteer

Home Visit Booklet


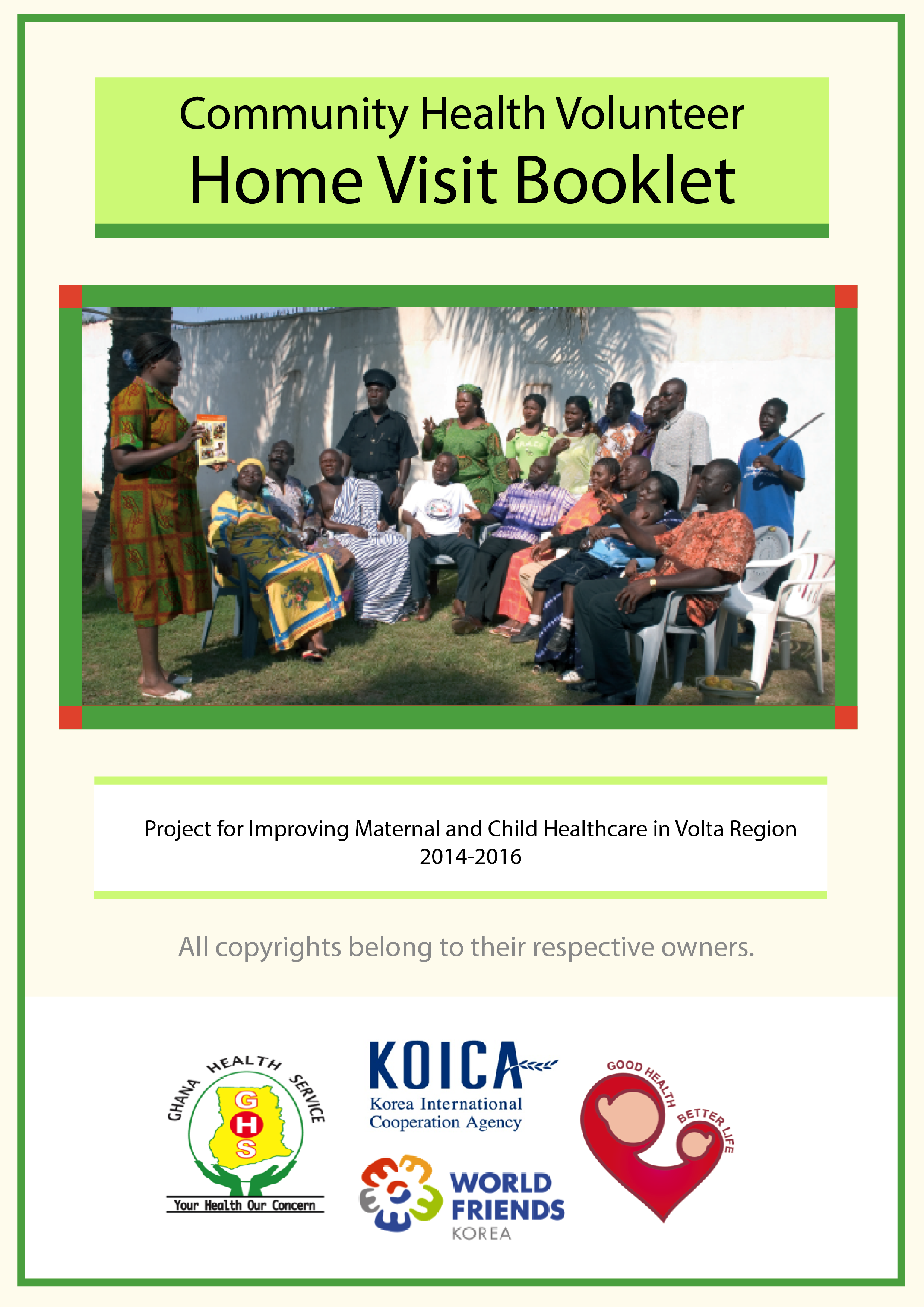


Project for Improving Maternal and Child Healthcare in Volta Region

2014 – 2016

| 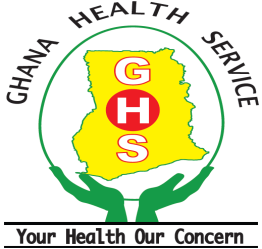 | 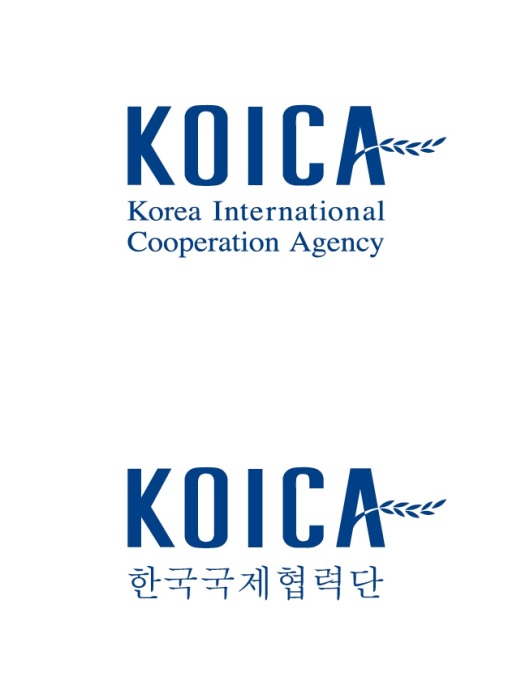 | 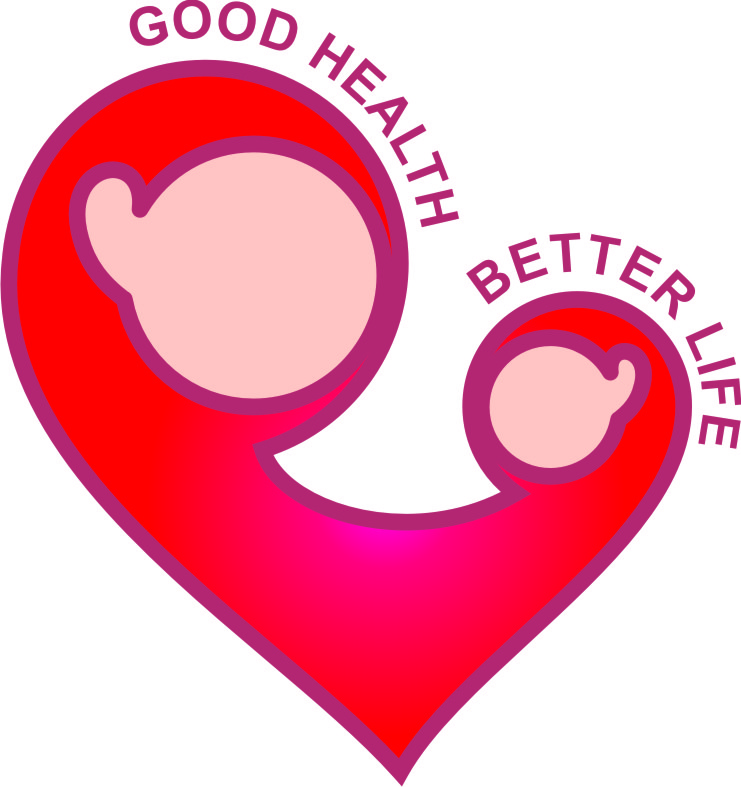 |
| --- | --- | --- |

All copyrights belong to their respective owners.

**KEY MESSAGE**

**(1) Family planning**

1. Family planning prevents unwanted pregnancy.
2. Go to health facility for counseling to find the best choice for each woman.
3. Condoms can be always used alternatively.

**(2) Antenatal Care**

1. Visit the health facility and attend ANC when you get pregnant.

**(3) Delivery by Skilled Birth Attendant**

1. Delivery at health facility keeps you and your baby safe and healthy.

**(4) Postnatal Care**

1. Visit the health facility with your baby to attend PNC immediately after an unexpected delivery in the house.

**(5) Exclusive breastfeeding for 6 months**

1. To keep your baby healthy, practice exclusive breastfeeding for 6 months.

**(6) Prevention of diarrhea in under-5 children**

1. Proper hand-washing and clean latrine prevents diarrhea.
2. Mothers must wash their hands with soap under running water at 5 critical moments:

1)before cooking, 2)before eating, 3)before feeding, 4) after handshaking, 5)after defecating (visiting the toilet).

1. Avoid open defecation and keep the latrine clean to avoid flies.

**(7) Management of diarrhea**

1. Use ORS and Zinc tablet and immediately go to the health facility for treatment.
2. If ORS is not available, use rice water, coconut water or mashed kenkey.

**(8) Prevention of malaria**

1. Every person, especially pregnant woman and child under-5 should sleep under ITNs (Insecticide-treated nets).
2. Pregnant women should take SP (IPT) to prevent malaria.
3. People diagnosed positive to malaria should go to health facility for treatment.

**(9) Prevention of anaemia**

1. Eat green vegetables and fruits.
2. Visit health facility to take iron drugs.
3. Deworm your children every 3 months.

**(10) Participation in CWC (Child welfare clinic)**

1. Go to CWC every month for weighing and vaccinating your children up to 5 years.
2. **Family planning**


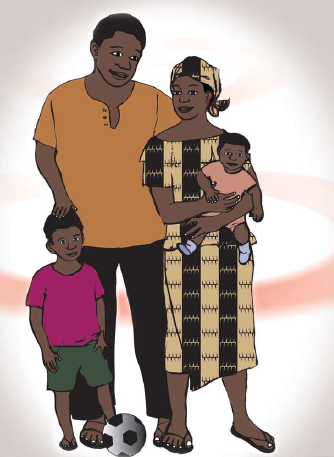


| 1. **Family planning prevents unwanted pregnancy.** 2. **Go to health facility for counseling to find the best choice for each woman.** 3. **Condoms can be always used alternatively**. |
| --- |

1. **Benefits of Planning your family**
2. Helps couples/ partners have the number of children they want when they want
3. Helps improve the health of mothers and children through child spacing can help couples/partners plan better for their children’s needs like food, clothing, education


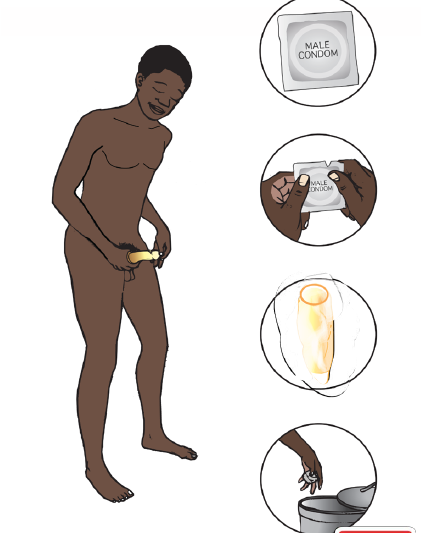


1. **Male condom**

**Advantage**s

1. It is safe and

effective

1. It is convenient ,

easy to use and

affordable

1. It can prevent

both pregnancy

and STIs(e.g

Gonorrhea, Syphilis,

HIV)

**Disadvantages**

1. Need to use one condom for each sexual act
2. Some people (both men and women) may be allergic to the latex(rubber)
3. Condom may break or slip off

**How to it works**

1. The condom should be removed after sex before the penis loses its erection
2. It should be properly disposed of after use(put it latrine, or buried)
3. **Oral Contraceptive Pill**

**
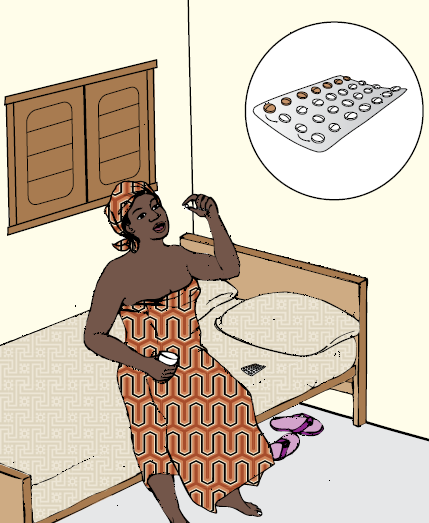
**

**Advantages**

1. The Pill is safe and very effective
2. The Pill can be taken by most women from menarche to menopause
3. It is reversible and return to fertility is rapid once women stops taking the Pill
4. It does not interfere with sexual intercourse

**Disadvantage**

1. The Pill must be taken **everyday** therefore may be easy to forget
2. It does not protect against STIs, including HIV
3. **The Injectable**


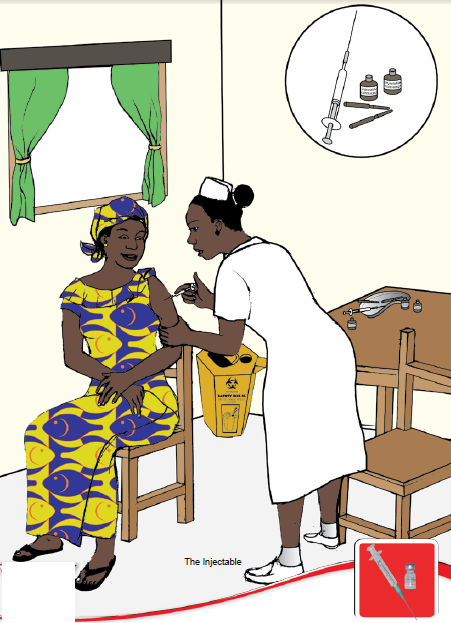


**Advantages**

1. It is very effective and safe
2. It is easy to use and reversible
3. It does not interfere with intercourse
4. **Implants**

**
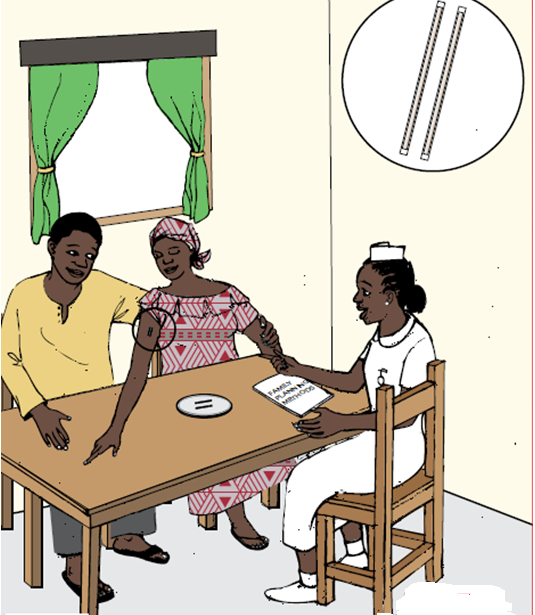
**

**Advantages**

1. Implants are safe and immediately effective(within 24hours)
2. Return to fertility is immediate once the Implants are removed
3. They can be taken out anytime, if the woman desires to become pregnant or for any other reason

❖ A trained health provider is need to insert and remove Implant

1. **Family planning after child birth**


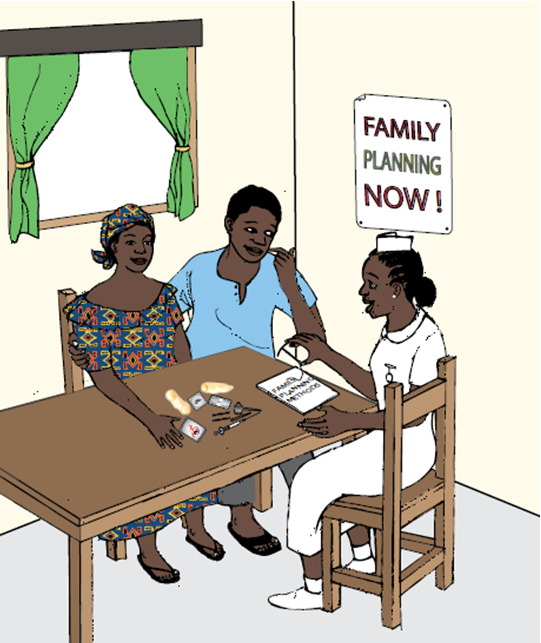


After delivery, miscarriage or abortion all mothers should be encouraged to use an effective family planning method

**Benefits of Spacing pregnancies;**

1. The baby can breastfeed longer and avoid growth problems
2. The mother can regain her strength
3. The mother can avoid stress and problems of a new pregnancy
4. The Mother will have time for the baby
5. The next baby is likely to be born healthy and strong
6. **Antenatal Care**

**
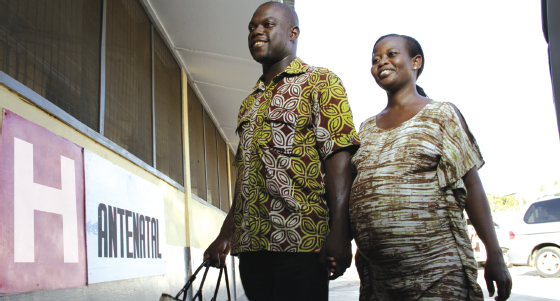
**

| 1. **Visit the health facility and attend ANC when you get pregnant** |
| --- |

1. **Benefits of Antenatal care**
2. ANC is reducing maternal and perinatal complication
3. It has been estimated that 25 percent of maternal deaths occur during pregnancy, due to causes such as hypertension and antepartum haemorrhage

1. **When to visit the health facility for ANC**
2. All women with uncomplicated pregnancy should have a minimum of four visits
3. Booking During first trimester and preferably

Before 14 weeks

1. 1^st^ visit End of fourth month

(Preferably at 16 -20 weeks)

1. 2^nd^ visit Around sixth month

(At about 24 weeks)

1. 3^rd^ visit In eighth month

(28-32 weeks)

1. 4^th^ visit In ninth month

(About 36 weeks)

1. More frequent visits or different schedules are based on the woman’s individual needs
2. Remind pregnant women to keep their appointment

**3. Delivery by Skilled Birth Attendants**

| 1. **Delivery at health facility keeps you and your baby safe and healthy** |
| --- |

1. **Benefits of delivering by a skilled birth attendant**
2. Takes appropriate care of the cord
3. Manage a cord around the baby’s neck at delivery
4. Clamp and cut the cord using aseptic technique
5. Check the placenta and membranes for completeness
6. Manage postpartum haemorrhage

**4. Post natal Care**

| 1. **Visit the health facility with your baby to attend PNC immediately after an unexpected delivery in the house** |
| --- |

1. The days and weeks following childbirth – the postnatal period – is a critical phase in the lives of mothers and newborn babies. Most maternal and infant deaths occur during this time.
2. **Number and timing of postnatal contacts**
3. If born in the health facility: the first postnatal contact should be in 6-7 days after birth
4. If birth is at home, the first postnatal contact should be as early as possible within 24 hours of birth (***within 2 days of birth****).*
5. At least three postnatal contacts are recommended for all mothers and newborns
6. On day 3 (48–72 hours)
7. Between days 7–14 after birth (Register birth of the baby)
8. At six weeks. Register birth of the baby
9. **Danger signs of the baby**

1. The family should be encouraged to seek health care early if they identify any of the danger signs in-between postnatal care visits
2. Stopped feeding well
3. History of convulsions
4. Fast breathing (breathing rate ≥60 per minute)
5. No spontaneous movement
6. Fever (temperature ≥37.5 °C), low body temperature (temperature <35.5 °C)
7. Any jaundice in first 24 hours of life, or yellow palms and soles at any age
8. **Postnatal care for the newborn**
9. **Cord care :** Dry and do not apply anything on the cord
10. **Bathing** : should be delayed until 24 hours after birth or at least six hours
11. Appropriate **clothing** of the baby for ambient temperature is recommended.

One to two layers of clothes more than adults, and use of hats/caps

1. Immunization should be promoted

**5. Exclusive breastfeeding for 6 months**

Only breast milk, and no other foods or liquids

| 1. **To keep your baby healthy, practice exclusive breastfeeding for 6 months.** |
| --- |

1. **Benefit of breastfeeding for the baby**
2. Clean, because it does not become contaminated by dirty hands, spoons, cups and flies
3. Always available and requires no special preparation
4. Provides the ideal [nutrition](http://www.webmd.com/diet/default.htm) for infants
5. It is a form more easily digested than [infant](http://www.webmd.com/parenting/baby/default.htm) formula
6. Contains antibodies that help your baby fight off viruses and bacteria
7. Babies who are breastfed exclusively for the first 6 months, without any formula, have fewer [ear infections](http://www.webmd.com/cold-and-flu/ear-infection/default.htm), respiratory illnesses, and bouts of [diarrhea](http://www.webmd.com/digestive-disorders/digestive-diseases-diarrhea)

**6. Prevention of diarrhea in under-5 children**

| 1. **Proper hand-washing and clean latrine prevents diarrhea.** 2. **Mothers must wash their hands with soap under running water at 5 critical moments: 1) before cooking, 2) before eating, 3) before feeding, 4) after handshaking, 5) after defecating (visiting the toilet).** 3. **Avoid open defecation and keep the latrine clean to avoid flies** |
| --- |

1. **Definition of Diarrhoea**
2. Diarrhoea is a condition in which the stools contain more water than normal. The stools are loose or watery
3. It is defined as passing three or more loose or watery stools in a 24 hours period
4. Babies who are exclusively breastfeed often have stools that are soft: this is not diarrhea
5. **Risks of diarrhea in children**
6. Lose water from the body quickly (*dehydration*)
7. Lose energy that they need to fight disease and for growth (*malnutrition*)
8. Lose important substances in the body that help the body to work correctly
9. All these can quickly lead to death of the child
10. **Cause of diarrhea**
11. Eating food that contains germs (the food may look normal)
12. Drinking water that contains germ
13. Eating food or feeding children without washing your hands
14. Feeding children with dirty utensils (such as plates, cups and spoons)
15. **Prevention of diarrhea;** Parents/caregivers should
16. Wash hands before feeding the child or wash child’s hand before she/he eats
17. Practice exclusive breast feeding for children less than 6 months
18. Wash fruits or vegetables thoroughly before the child eats them
19. Give the child clean water
20. Cover all dustbins and properly dispose all refuse in a pit
21. Give ORS to all children with diarrhea
22. **Hand washing**
23. When to wash your hands

| **Before** | **After** |
| --- | --- |
| 1. Preparing food 2. Eating 3. Treating wounds or cuts 4. Touching sick or injured person 5. Feeding children 6. Preparing ORS 7. Giving medication | 1. Preparing food 2. Eating 3. Treating wounds or cuts 4. Touching sick or injured person 5. Using the toilet 6. Touching animals or animal waste 7. Blowing your nose 8. Handling refuse or garbage 9. Changing the child’s napkin 10. After coughing or sneezing into your hands |

1. **How to wash one’s hands**
2. Wet hands with running water
3. Rub hands together with liquid soap or bar soap to lather well
4. Scrub all surfaces, including the inner and backs of the hand, the wrists, between the fingers, and under your fingernails
5. Rinse well under clean running water until all soap is gone
6. Dry hands with clean towel or allow hands to dry in the air

| 1. **Avoid open defecation and keep the latrine clean to avoid flies**. |
| --- |

1. If you do not have a latrine, you should dig before defecation and bury afterwards. It is only recommended when there is no latrine accessible.
2. Open defecation is a leading cause of diarrheal death and it also causes cholera, intestinal worm infections, typhoid, hepatitis A etc.


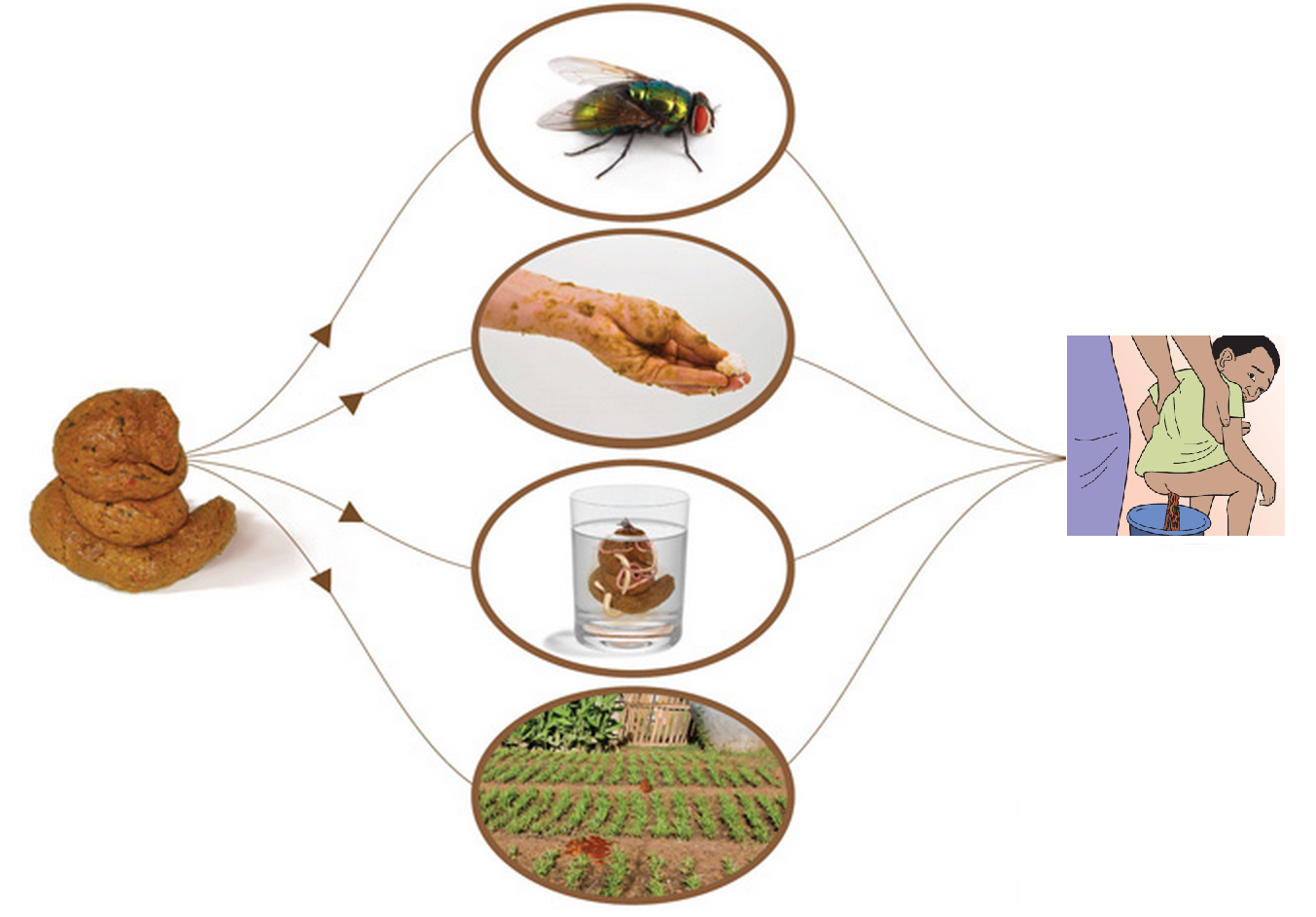


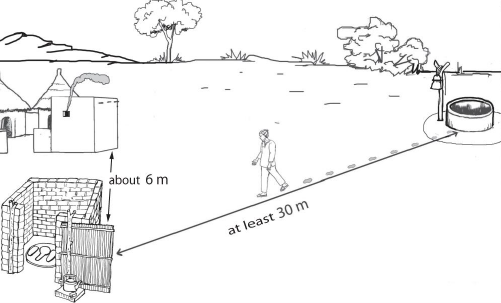


1. Latrine must be placed at least 30m from a well and about 6m from a kitchen

**

7. Management of diarrhea**

| 1. **Use ORS or Zinc tablet and immediately go to the health facility for treatment.** 2. **If ORS is not available, use rice water, coconut water or mashed kenkey.** |
| --- |

1. **Mixing Oral Rehydration Salts**


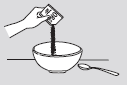

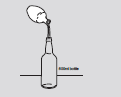

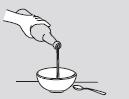


1. Check Expiry date
2. Wash your hands with soap and water.
3. Measure 500ml; one big beer bottle or 2 small fanta bottles of water from the tap or borehole and pour into a clean cup or container with a lid
4. If water is not from tap or borehole, boil and cool it before measuring it.
5. Tear open one sachet of ORS and pour into the water in the bowl, stir with spoon to dissolve.
6. Serve in smaller quantities at frequent intervals until solution is finished.

Taste the solution, it should taste a little bit salty, like tears.

1. Do not use the drink after 24 hours

1. **To give ORS**
2. How
3. Give frequent small sips from a cup or spoon
4. If the child vomits, wait 10miutes and then continue but more slowly
5. Continue giving extra fluids until the diarrhoea stops
6. How much
7. Give extra fluids ( in addition to how much the child usually takes)
8. Give at least the following amounts:

Less than 2 years: 50 to 100ml after each loose stool

2years or more: 100 to 200ml after each loose stool

1. If the parent/caregiver cannot measure the exact amount as, advise him/her to give the child as much ORS as the child can take and at short intervals
2. **Home treatment of Diarrhoea**

For effective treatment of diarrhea, it is very important to follow four basic rules

1. Give extra fluid(as much as the child will take)
2. Give Zinc supplements daily for 14days
3. Even after diarrhoea stops
4. For infants : dissolve tablet in a small amount of expressed breast milk, ORS or clean water in a cup
5. For older children: tablets can be chewed or dissolved in a small amount of clean water in a cup

|  | Zinc 10mg tablet | Zinc 20mg tablet |
| --- | --- | --- |
| Age  6 months up to 5 days | Daily for 14days | Daily for 14days |
|  | 2 | 1 |

1. Continue feeding(as much as child will take)
2. Feed small amounts and frequently
3. Offer the child his or her favorite foods
4. If the child refuse to eat, wait a little and try again
5. *Go directly to the CHO/Clinic*
6. If the child does not better in 2 days
7. If the child gets worse
8. If the child has ANY DANGER SIGN

**8. Prevention of malaria**

**
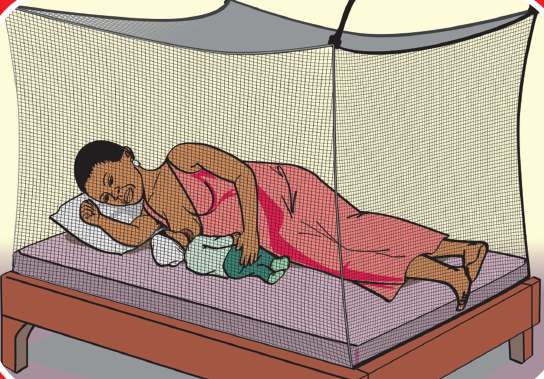
**

| 1. **Every person, especially pregnant woman and child under-5 should sleep under ITN(Insecticide-treated net)** 2. **Pregnant women should take SP(IPT) to prevent malaria.** 3. **People diagnosed positive to malaria should go to health facility for treatment.** |
| --- |

1. Malaria-carrying mosquitoes breed in clean, still or slow moving water, such as in puddles, empty cans, used tyres, flower pots, ponds, rice fields, streams, etc.

Removing standing water in and around your house can help to reduce the number of mosquitoes, but will have little effect on reducing malaria

1. **Benefits of Prevention**
2. Healthy pregnancy and newborns. Pregnant women will be more likely to:
3. Carry the pregnancy to full-term and avoid miscarriage
4. Give birth to a healthy at normal weight
5. Better child development
6. Save money. As a family the money you would have spent on malaria medicines and transport to/from clinic can go to things like education, food, entertainment and the like
7. Make more money, be more productive. You will not miss work days due to malaria, and you will be able to complete more work whether on your farm, fishery workplace, office or home


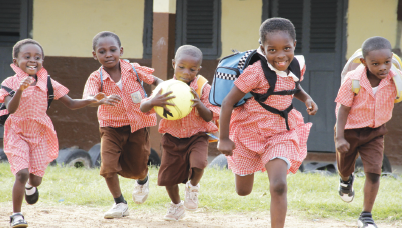


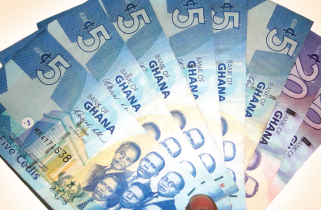


| 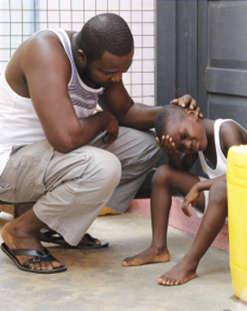  Headache | 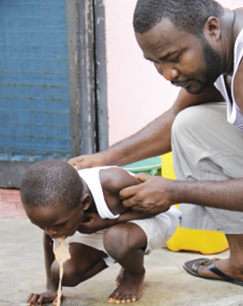  Vomiting |
| --- | --- |
| 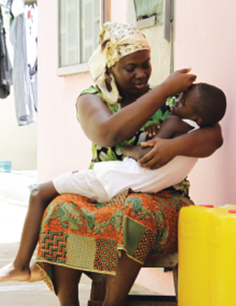  Fever | 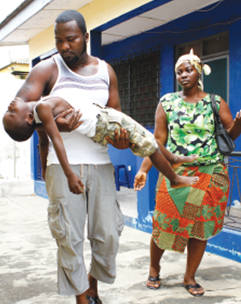  Convulsion |

1. **Malaria signs and symptoms**
2. Uncomplicated Malaria; if not treated early and completely, it can quickly turn into severe malaria
3. Severe Malaria is a life-threatening condition.

It is an emergency and you need to rush the patient to a health facility immediately.


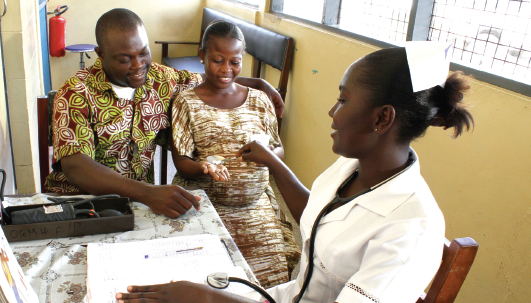


1. **Malaria in Pregnant Women**

A pregnant women is more at risk from malaria because her body is working hard to grow the baby.

1. Miscarriage, spontaneous abortion and still birth(death of the unborn child before or at delivery)
2. Maternal anaemia
3. Low-birth weight newborn babies(small, unhealthy babies) and Preterm birth
4. **Prevent of malaria in pregnant women**
5. Sleep under a treated net every night
6. Go to the health facility for antenatal care as soon as you know/feel you are pregnant
7. Take iron supplements and eat well during the pregnancy, including lots vegetables, fruits and proteins


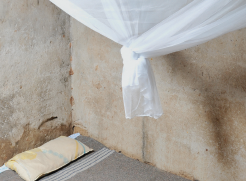

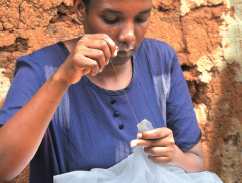


Tie up net to prevent damage Sew holes shut if net tears


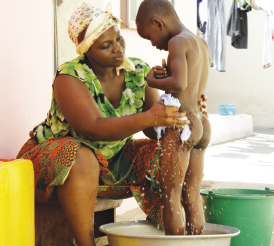

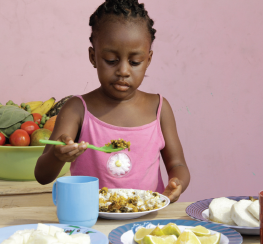


1. How to use your Treated Net
2. Hang the net over your bed or sleeping area using nails or hooks and strings
3. At bed times, lower the net to cover the sleeping area. Tuck the edges under the mat trees or mat so mosquitoes cannot enter
4. At day, tie up the net to prevent it from being damaged or torn
5. **How to care for your Treated Net**
6. Gently wash your dirty net with plain water. You may use some mild bar soap if necessary, but never use powdered detergent. Dry in the shade. Do not place it in direct sunlight
7. If the net is torn or has holes, sew the holes shut as you would any other fabric
8. Treated Net can be gently washed up to 20 times. It will last for 4 years if you wash it 5 times per year
9. **Early and Complete Malaria Treatment**
10. Start treatment early to avoid severs malaria
11. As soon as you suspect malaria, take children under 5 years old and pregnant women to the health clinic immediately
12. Complete full dose
13. If you do not complete the treatment, the malaria will come back and could be more severe
14. Do not stop taking the drugs, if you feel better. Keep taking it each day until the dose is completed
15. **Malaria in Children**
16. Children are not yet strong as adults and are still growing and developing
17. Children can die or suffer lifelong health problems within 24 hours of developing malaria if not treated early

**9. Prevention of anemia**


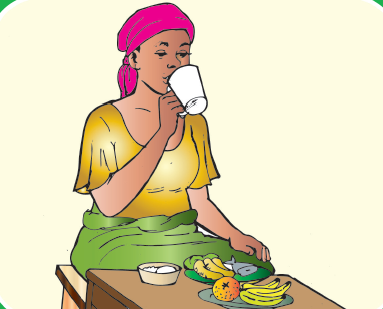

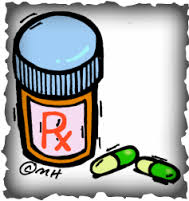


| 1. **Eat green vegetables and fruits.** 2. **Visit health facility to take iron drugs.** 3. **Deworm your children every 6 months;** For children 2 years and above |
| --- |

1. **Cause of Anaemia**
2. Poor nutrition: lack of balanced diet and iron
3. Malaria: malaria parasites kill red blood cells. Repeated episodes of malaria that are not completely treated makes anaemia more severe
4. Worms: worms prevent nutrition from getting to the body
5. Infections: diarrhea and pneumonia weaken the person
6. **Effects of anaemia**
7. Poor learning ability, developmental and growth problems in children and unborn babies including brain damages and weak physical development
8. Weak, tired feeling all the times no matter how much sleep you get
9. **Foods for anaemia**
10. Iron-rich foods; meat, chicken, fish and dark green vegetable – and fruits


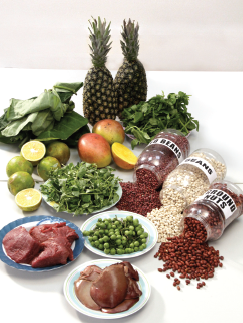

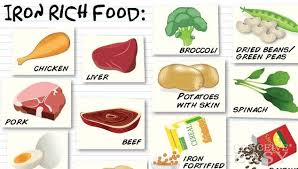


1. **Foods for good nutrition**
2. **Energy-giving foods** burn in the body and provide us with energy for moving, sleeping, working, etc.
3. **Body-building foods** provide nutrients for building the muscle, bone, skin, brain and hair
4. **Protective foods** are vitamins and minerals: they protect our eye, skin etc. from infection and disease


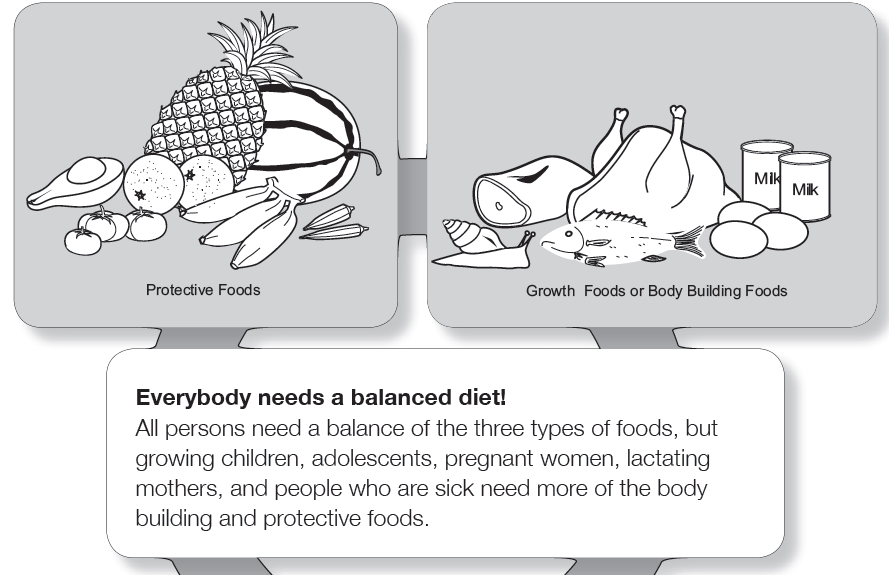


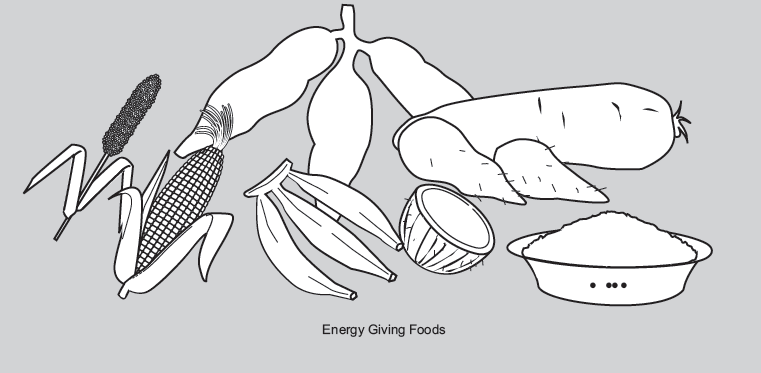


| 1. **Deworm your children every 6 months;** For children 2 years and above |
| --- |


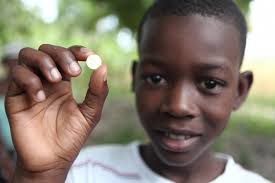

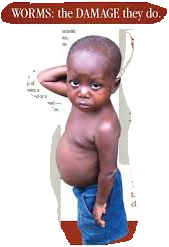


1. Worms are parasites that inhabit several places including the human body

Worm infestations can present with diarrhoea, loss of appetite, and abdominal cramps

1. **Benefits of Prevention**
2. Prevents malnutrition such as stunting, wasting
3. Prevents iron-deficiency anemia
4. Have a positive health effect in long-run
5. Health sustainable opportunity of education (Deworming increases school attendance.)
6. Even untreated children can benefit from deworming.

**10. Participation in CWC (Child welfare clinic)**


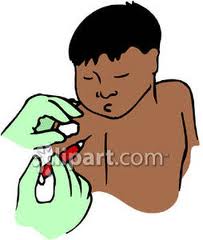

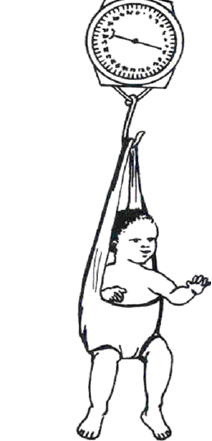


| 1. **Go to CWC every month for weighing and vaccinating your children up to 5 years.** |
| --- |

All copyrights belong to their respective owners.

1. Home based maternal and newborn care (Care of the mother and baby

through home visits): Home Visit Booklet.

Developed by Ghana Health Service, KOICA, UNICEF and Japan Official Development Associate. June 2014.

1. Drive malaria away for good life.

Developed by Ghana Health Service, USAID, BCS Project, Pro MPT Ghana.

1. Family Planning Flip Chart.

Developed by Health Promotion Department of Ghana Health Service. 2012.

1. Home management of malaria, acute respiratory infection & diarrhea

in Ghana: Manual for Community based agents.

Developed by Ministry of Health, Ghana Health Service, The Global Fund, President’s Malaria Initiative, CHPS-RA. March 2009.

1. How to build your latrine and use it hygienically, for the dignity, health, and well being of your life: Practical guide for building a simple pit Latrine. Developed by GWI West Africa. June 2011

► For questions or more information please contact:

| **KOICA (Korea International Cooperation Agency) MCH Project Office**  2^nd^ Low Cost Road – Aflao  Tel: 036 253 10 050 |
| --- |
