# Supplementary material for: Effects of community health volunteers on infectious diseases of children under five in Volta Region, Ghana: study protocol for a cluster randomized controlled trial
Source: BMC Public Health. 2017 Jan 19;17:95. doi: 10.1186/s12889-016-3991-z (PMC5244532; doi:10.1186/s12889-016-3991-z)
Supplement: Additional file 4: — Logical framework. (DOCX 24 kb) [file 12889_2016_3991_MOESM4_ESM.docx]

Table 1 Logframe

| Objectives | Measurable indicators | Means of verification |
| --- | --- | --- |
| Goals:  Sustainable improvement in prevention and management of infectious disease in children under five through activation of CHV | Number of malaria and diarrhea deaths of children under five | Annual/Half-year reports of district health service |
| Purposes:  1. Prevention & management of child diarrhea  1.1 Reduction of diarrhea prevalence in children under five is reduced.  1.2 Increase in the children under five with proper diarrhea management  2. Prevention & management of child malaria  2.1 Reduction of fever prevalence in children under five is reduced.  2.2 Increase in the children under five with fever getting malaria test for diagnosis | **(main indicators):**  1.1 7-day prevalence of diarrhea in children under five  1.2 Proportion of children under five with diarrhea administered ORS  2.1 14-day prevalence of fever in children under five  2.2 Proportion of children under five with fever tested using a RDT kit | Household survey  Household survey  Household survey  Household survey |
| Intermediate outcomes:  1. Improved behavior of caregivers in preventing diarrhea  2. Improved behavior of caregivers in preventing malaria | **(Intermediate indicators):**  1.2. Proportion of caregivers who perform hand-washing at 4 critical times^[[1]](#footnote-1)^  1.2. proportion of households treating water properly  1.3 proportion of households utilizing latrine  1.4 proportion of households cleaning latrine regularly  1.5 proportion of households disposing child feces properly  1.6 proportion of children under six months exclusively breastfed  2. Proportion of caregivers who utilized insecticide bed nets for the child the previous night while sleeping | Household survey & observation  Household survey & observation |
| Outputs:  1. Improved awareness of caregiver in prevention and management of infectious disease in children under five | **(Process indicators):**  1.1 Proportion of caregivers who can recall key messages regarding prevention & management of diarrhea and malaria  1.2 Number of key messages recalled by caregivers | Household survey  Household survey |
| Activities:   - 1. Successful training and deployment of CHVs with qualification   2. CHVs’ regular home visits for health education using key messages and service delivery   3. CHV’s participation in monthly meetings for supervision and refresher training | **Inputs:**  Human resources (district health directorate staffs, CHNs, CHOs, project staffs)  Trainings   - Initial training for CHVs & CHOs - Monthly refresher training for CHVs   Guidelines and educational materials   - SOP for implementation of monthly meetings - Home visit booklet   Facilitative supervision   - Monthly visit by the district health directorate staffs and project staffs - Continuous supervision by CHOs   Provision of health equipment & supplies (ORS, zinc tablet, RDT kit, digital thermometer)  Material incentives for CHVs (food items & airtime) |  |

1. Before cooking, after defecating, before feeding the child, before eating [↑](#footnote-ref-1)
